# A multiple and comprehensive approach to assess health risk in amalgam-exposed Chinese workers

**DOI:** 10.3389/fpubh.2022.999095

**Published:** 2022-09-20

**Authors:** Xiao-ying Ruan, Si-wei Tan, Lin Zhu, Yan-peng Shi, Jia-mian Yu, Mei-bian Zhang, Tong-shuai Wang, Hong Fu

**Affiliations:** ^1^Department of Occupational Health, Hangzhou Hospital for Prevention and Treatment of Occupational Disease, Hangzhou, China; ^2^Department of Occupational Health, Chinese Center for Disease Control and Prevention, Beijing, China; ^3^Shanghai Key Laboratory of Maternal Fetal Medicine, Clinical and Translational Research Center of Shanghai First Maternity and Infant Hospital, Tongji University School of Medicine, Shanghai, China

**Keywords:** occupational health risk assessment, mercury, amalgam, fluorescent bulb manufacturing, multiple comprehensive methods analyze

## Abstract

Fluorescent lamp manufacturing workers have been extensively exposed to mercury (Hg). Our aim was to assess their health risks using several approved occupational health risk assessment methods, and to find out which method was more suitable for identification of occupational health risks. Work locations, and air and urine samples were collected from 530 exposed workers in Zhejiang, China. Based on the calculated exposure doses, health risks and risk ratios (RRs) as health risk indices, were evaluated using: Environmental Protection Agency (EPA), Australian, Romanian, Singaporean, International Council on Mining and Metals (ICMM), and Control of Substances Hazardous to Health (COSHH) methods. Among the workers, 86.0% had higher Hg levels than the Chinese occupational exposure limits of 0.02 mg/m3, and 16.7% urine samples were higher than the biological exposure limits of 35.0 μg/g·creatinine. Among workers at the injection, etc. locations, their average RRs, evaluated by the EPA, COSHH and Singaporean methods were 0.97, 0.76, and 0.60, respectively, and were significantly higher than the ICMM (0.39), Australian (0.30) and Romanian (0.29) methods. The RRs from the Singaporean method showed significant correlations with the urinary Hg levels (*P* < 0.01). In conclusion, the Singaporean method was more appropriate than the others for health risk evaluation because the excessive risks were significantly associated with urinary Hg levels among the workers.

## Introduction

Incandescent bulbs have been widely replaced by fluorescent lamps (FLs) in order to solve the global problem of power shortages (1). Currently, a large portion of FLs has been manufactured in Asia, especially China, employing an enormous number of workers (2). However, each fluorescent lamp needs to contain a certain amount of mercury (Hg) to work properly. To reduce Hg health hazards and pollution to the environment, liquid Hg was used *via* its transformation into the solid form (e.g., amalgam) for FL manufacturing. Therefore, the underlying health effects of mercury to workers have been raising more and more concerns.

Amalgam is a solid mixture in which Hg atoms or fine Hg particles were adsorbed onto a carrier. Although Hg in amalgam is stable at room temperature, it can be evaporated readily at high temperatures, e.g., during Hg injection in FL manufacturing when the vapor pressure of amalgam becomes similar to that of liquid Hg. Hence, FL workers have been exposed to Hg which is ranked in the top three potent toxic agents by the Environmental Protection Agency (EPA) (3, 4). Indeed, excessive inhalation of Hg vapors caused damage to the digestive, immune, urinary, and nervous systems (5–7). A few of studies (8, 9) have assessed exposure biomarkers of Hg, such as blood mercury and urine mercury among occupational Hg-exposed workers. Although these reports identified excessive exposures, the information cannot be readily converted into health risk assessments. Thus, there is an urgent need to identify an appropriate assessment method that can be performed to evaluate the mercury-related health risks.

Regarding the FL manufacturing process, exposure to Hg was reported to be excessive, especially in the process of exhausting, basing, sealing and lamp assembling (10–12), e.g., Hg-vapor concentrations ranged from 23 to 175 μg/m3 in the air and 80% of the workplaces exceeded 25 μg/m^3^, which exceeded the American Conference of Governmental Industrial Hygienists' (ACGIH) occupational exposure limit (OEL) standard. Additionally, many workers had 44.1 ± 17.5 μg/g·creatinine in urine which was much higher than the biological exposure limit (BEL) standard of ACGIH at 20 μg/g·creatinine. Unfortunately, no health assessments, such as using OHRA, have been reported especially in using air concentrations and/or exposure biomarkers. Thus, the hypothesis for our investigation was that excessive exposures would be significantly associated with results from health risk assessments.

Despite excessive exposure to Hg among FL workers, especially in China, their health risks have not been adequately investigated (13). Indeed, their health risk can be assessed using different international occupational health risk assessment (OHRA) method, the semi-quantitative EPA method (14), the COSHH method (15), the Australia method, the Romania method, and a Semi-quantitative ICMM method (16). Each method had its own pros and cons based on their unique methodological principles; therefore, a combination of multiple methods would be advantageous for developing more reliable risk assessment and disease prevention strategies (17). In our investigation, these methods were used together with a comprehensive determination of exposure doses.

A total of 7 billion FLs were manufactured in China, accounted for over 80% of the world-wide production and the process consumed 29.31 tons of Hg (18, 19). Many FL manufacturers have been located in the Hangzhou in China and the province has one of the highest emissions of Hg in China (20, 21). Therefore, our health risk assessment investigation, using several international assessment methods, was conducted in the Zhejiang province to seek a reliable and rational way to evaluate the health risks of Hg-exposed workers. Based on our knowledge, our investigation is the first in China using these methods in a large group of amalgam-exposed workers.

## Materials and methods

### Subjects and exposure conditions

The common procedures for FL production involve the following steps: (1) Coating—coat the inner surface of glass tubes with a phosphor mixture by machine and sent for drying, (2) Sealing—seal the dried glass tubes with discharge tubes which contained metal electrodes inside, (3) Injecting—inject the amalgam into the glass tubes, pump in argon and apply vacuum before installation of pedestals, (4) Testing—test and adjust FLs to specified values (voltage, time, etc.). After quality testing, the products are packed and stored.

According to the occupational health supervision and management system, all 530 workers with excessive amalgam exposure conditions from different factories in Hangzhou were recruited based on the OHRA core steps during 2017 to 2020 and assigned into different groups: workers within similar exposure groups (SEGs). The inclusion criteria of workers included: (1) Exposed to amalgam for at least 2 months, (2) Not been absent within the past month, (3) Had no previous occupation-related diseases. The SEGs were categorized by the types of work: venting, amalgam injection, sealing, products testing, and etc. **Table 2** lists the general information for SEGs at different key positions. To further confirm exposures, Hg-exposed workers were selected based on an >50 h per week and they were divided into semi-automatic and manual operations groups. In their workplaces, exhaust ventilation systems were installed at key working stations and personal protective equipment (PPE) was available for workers. This work was approved for research from Hangzhou Hospital for Prevention and Treatment of Occupational Disease (IRB: 2020-001).

### Hg exposure assessment

Levels of Hg in the air were measured at each work location. Based on field investigations, the 8-h time-weight average concentrations (PC-TWA) were used as observation indices to evaluate the atmospheric exposure concentrations for SEGs. Human samples were taken from both urine and feces from workers. Urinary Hg (U_Hg_) level was chosen to be an important biomarker in the study since it can reflect cumulative doses of Hg. In addition, it can be used to verify results from risk assessment. The methods for sample collection and laboratory analysis are described below:

#### Air samples

Air samplings were performed according to the national standard of China (GBZ 159-2004). The combination of area and short-time samplings were used. Work sites with Hg exposure were selected as sampling points, and air extractors were placed as high as the standard human breathing line. Air samples were taken at each of three different work-shifts in a day, which were 7:30–10:30 a.m., 10:30 a.m. to 2:30 p.m. and 2:30 p.m. to 5:30 p.m. The air flow was set at 500 mL/min for no more than 15 min. One hundred and twenty-eight jobs were involved and 384 samples were taken. The C_TWA_s (the 8-h time-weight average concentration of Hg in the workplace air) were calculated using Equation (1):


(1) $$\begin{array}{l} C_{\text{TWA}}=\left(C_{1}\times T_{1}+C_{2}\times T_{2}+C_{3}\times T_{3}\right)/8 \end{array}$$

Where: C_1_, C_2_, and C_3_ means detected Hg concentrations in the workplace air (μg/m^3^), T_1_, T_2_, and T_3_ means working hours of workers at a corresponding Hg concentration (h), 8 means the permissible concentration-time-weight average was set at 8 h.

Laboratory tests of air samples were conducted according to the national standard: Hg values of workplace air samples were determined by atomic fluorescence spectrometry (Haiguang AFS-9560, Beijing) with a set of 193.7 nm Hg atomic absorption wavelength and atomic fluorescence intensities were measured by peak heights and peak areas.

#### Biological samples

Collections and tests of urinary Hg were based on Determination of Mercury in Urine-Cold Atomic Absorption Spectrometric Method (II) Acidic Stannous Chloride Reduction Method (WS/T 26-1996). After-work urine samples were collected in 100 mL polyethylene bottles, and specific gravities of urine were measured on the same day. To determine the concentration of Hg in human urine samples, sulfuric acid and potassium permanganate were added into tubes of collected urines. The mixtures were placed into a water bath at 50°C to destroy organic substances which could change Hg into Hg ion. Furthermore, Hg ion was reduced to Hg by stannous chloride which was delivered into the Hg meter test tubes by air for measuring absorbance quantity. Each batch contained procedural blanks and replicate runs.

### Occupational health risk assessment (OHRA)

The collected Hg levels were evaluated using the occupational exposure limits (OELs) of PC-TWA (0.02 μg/m3) and the BEL in urine: (35 μg/g·creatinine). Based on their frequent use in published reports (16), the EPA, COSSH, Singaporean, Australian, Romanian, and ICMM methods were applied to investigate health risks among the Hg-amalgam exposed workers. Characteristics of each method are briefly summarized in Table 1.

**Table 1:** Brief characteristics of different methods.

**Method**	**Characteristics**	**Procedure**		
		**Step**	**Equations**	**Explanation**
EPA	Includes carcinogenic and non-carcinogenic risk evaluations. In this study, non-carcinogenic risk assessment was conducted.	a) Estimating exposure concentrations (EC)	EC= (CA × ET × EF × ED)/AT (Equation 2)	Where EC (μg/m3) was the exposure concentration; CA (μg/m3) was the contaminant concentration in the air; ET (hours/day) was the exposure time; EF (days/year) was the exposure frequency; ED (year) was the exposure duration; and AT was the averaging time [ED (years) ×365 days/year ×24 h/day].
		b) Non-carcinogenic risk assessment	HQ = EC/RfC (Equation 3)	Where HQ was the hazard quotient; RfC was the reference concentration of inhalation toxicity; the limit of HQ was considered to be 1.
Singaporean	Risk levels were calculated based on hazard ratio (HR) and exposure ratio (ER).	Exposure concentration was available.	Risk = $\sqrt{HR\times ER}$ (Equation 4)	Where HR was assigned by the carcinogenicity classification of the International Agency for Research on Cancer (IARC). ER was based on the ratio of the exposure level (E) and permissible exposure limit (PEL) or OEL and represented the risk level of harm to human health from prolonged exposure to the chemical.
		Exposure concentration was not available, exposure indices (EIs) were used to determine the ERs.	ER= [EI_1_ × EI_2_ × …EI_n_]1/n (Equation 5)	Where EIs were determined by using exposure factors such as the vapor pressure, the hazard control measurements, the weekly amount, and the weekly duration.
COSHH	This method used a generic risk assessment approach to recommend the control levels. Health hazard was determined based on allocating of the evaluated substance to a hazard band and a Risk-phrase was given. Exposure potentials were determined by allocating the substance to an appropriate band, for an instance, dustiness, volatility or scale of use.			
ICMM	The matrix method was applied to assess risk levels in this method. The matrix included a combination of health hazards, possibility of occurrence and exposure levels.			
Australian	Risk levels were assessed by using a manual diagram to analyze identified exposure levels, possible consequences, and the likelihood of exposure.			
Romanian	Risk levels were qualitatively estimated by the matrix method. Based on the severity and occurrence of hazard, risk acceptability curve was also illustrated.			

#### Risk ratio calculations

Comparisons between different methods were performed based on analyses of risk ratios (RRs). The RR was the ratio between the risk levels and the maximum risk levels of a particular risk factor, which made the relative risk levels from different methods comparable. For example, the risk level of Hg in the Singaporean method at the venting location was 4, while the maximum risk level was 5, thus the RR was 0.8 (4/5). The calculations of RR for the EPA and COSHH methods were based on the classification criteria of the Singaporean method. In the Singaporean method, four specific cut points (×0.1, 0.5, 1.0, 2.0) of E/PEL were used to categorize the exposure ratings (ER). Total risk levels were calculated based on the levels of ER and HR. Generally, the E/PEL ×0.1 and E/PEL ×0.5 were considered as the safety and action levels, respectively, by the NIOSH and OHSA, USA. Thus, the risk levels (<1, >1) of hazardous quotient (HQ) used in the non-carcinogenic evaluation of EPA method were re-categorized (<0.1, 0.1–0.5, 0.5–1.0, 1.0–2.0, and >2.0). Meanwhile, the control strategy levels of 2, 3, 4 in the COSHH method were equivalent to the risk levels of 3, 4, 5 based on a comparative study (22).

#### Verification of hazard ratios (HR)

Data obtained from the six methods were evaluated and compared according to the Toxicant Hazardous Index (THI) of Globally Harmonized System of Classification and Labeling of Chemicals (GHS) and Chinese National Standard of Hazards Classification for occupational exposure toxicant (GBZ 230-2010). THI was determined using the acute inhalation toxicity (median lethal concentration, LC_50_), acute dermal toxicity (median lethal does, LD_50_), corrosion/irritation, sensitization, reproductive toxicity, carcinogenicity, actual hazard consequence and prognosis, diffusivity and accumulation, and other GHS-related indicators. Integral value (*F*) and the weight coefficient of sub-index (*k*) were quoted from GBZ 230-2010, and then calculated based on Equation (6). The related toxicity information was from the US Hazardous Substances Database (HSDB) and the Registry of Toxic Effects of Chemical Substances (RTECS).


(6) $$THI=\sum_{i-1}^{n}\left(k_{i}\cdot F_{i}\right)$$

The hazards classified into 5 grades: THI <20 (HR = 1), 20 < THI <35 (HR = 2), 35 < THI <50 (HR = 3), 50 < THI <65 (HR = 4), and THI > 65 (HR = 5).

#### Exposure ratios (ER)

Based on the grading method of the American Industrial Hygiene Association (AIHA) and 95% percentile of OEL, the exposure grading results were also compared with those obtained from the six methods. The exposure grades were organized into 5 levels: the 95% percentile <0.1 × OEL, ER = 1; 0.1 × OEL ≤ the 95% percentile <0.5 × OEL, ER=2; 0.5 × OEL ≤ the 95% percentile <1.0 × OEL, ER = 3; 1.0 × OEL ≤ the 95% percentile <2.0 × OEL, ER = 4; the 95% percentile≥2.0 × OEL, ER = 5. The OEL of Hg exposure by inhalation was 20 μg/m^3^ in China.

### Quality control design

To assure data quality, the following quality control measures were adopted: (1) Sampling and laboratory personnel were professionally trained; (2) Sample collection and inspection equipment have passed the metrology department verification, and calibrated according to relevant standards before use; (3) All reagents, filter membranes and other consumables were purchased from professional organizations with certificates, and used in accordance with the requirements of environmental conditions; (4) All data were double-checked and imported into SPSS 19.0 for data analyses.

### Statistical analysis

The skewed variables from the non-normal distribution data were expressed as the median (interquartile range, IQR), while the normal distribution data as the mean and standard deviation. One-way analysis of variance (ANOVA) was used for comparison between multiple samples, the LSD-t test was used for multiple comparisons when variances were equal, and the Dunnett T3 test was applied when variances were heterogeneous. The Spearman correlation test was used to analyze the correlations between the risk ratios obtained from different methods and the urinary Hg level of population. Correlations between biological indices (U_Hg_ values) and risk levels achieved from the six different methods were analyzed, and to further verify the evaluation results. The significance level (*P*-value) was set at 0.05.

## Results

### Fluorescent manufacturing industry in Hangzhou

**Figure 1:**
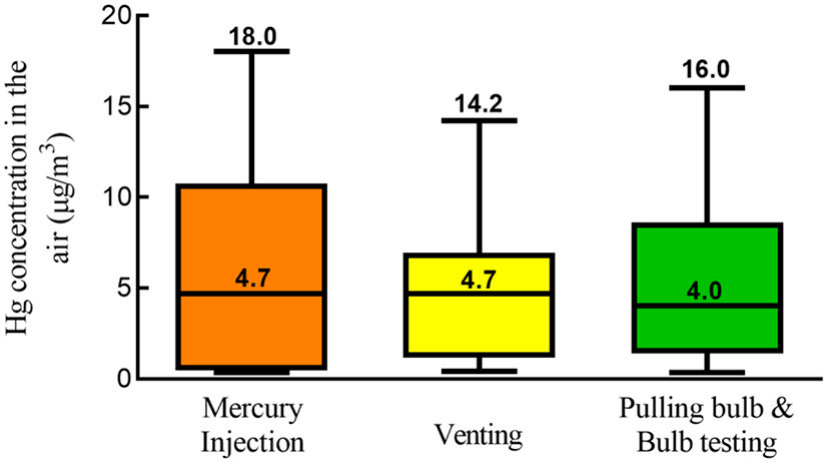
Distribution of mercury concentrations in different work locations.

According to the data filed by the occupational health supervision and management system of Hangzhou, the total number of workers in the FL industry was more than 5,000. Among them, more than 800 were directly involved in amalgam injection, accounting for 17.2% of the workers. In addition, these workers had daily exposure time between 7 and 8 h. The median and range of Hg concentrations among injection workers were 4.7 (0.4–18.0) μg/m^3^, among venting were 4.7 (0.4–14.2) μg/m^3^, and among pulling & testing were 4.0 (0.4–16.0) μg/m^3^. There was no significant difference in the Hg concentrations among the three positions (Figure 1). Employers indicated that, from occupational health examinations for these workers, there were no Hg toxicity nor occupational diseases. However, there was no specific effort to conduct health hazard evaluations.

### Characteristics of the study population

In total, 530 workers were recruited, with the mean age of 45.1 (SD 6.6) years old, with exposure duration of 1.8 (SD 1.3) years and with 40.6% of them being females. These workers were classified into three groups according to their work locations. Table 2 shows the work locations, the detection rate of mercury concentration in air was 86.0% while the over-standard rate was 0.9%, and the abnormal rate of mercury in urine of the population was 16.7%. Although Hg concentrations in air at the injection work site did not exceed the standard, the abnormal rate of urine Hg among the workers reached 66.7%. At the venting site, the abnormal rate of urine mercury was 7.69%, where the concentration of mercury in the air exceeded the standard rate of 1.6%. Except for the pulling and testing site, Hg concentrations in the injection and venting sites were higher than the overall levels in Hangzhou city.

**Table 2:** General information for similar exposure groups at key mercury (Hg) exposure locations.

**Work types**	**Workers**			**Air Hg concentrations (**μ**g/m**^**3**^**)**			**Urine Hg concentrations (**μ**g/g**·**creatinine)**	
	***N***	**Age**	**Exposure durations (year)**	**Median (range)**	**Exceeding detection limit^a^ (%)**	**Exceeding OEL^b^(%)**	**Medians (range)**	**Exceeding BEL^c^(%)**
Amalgam injection	122	48.3 ± 5.7	2.2	7.0 (0.65–17.0)	90.0	0	50.9 (12.6–89.4)	66.7
Venting	319	40.9 ± 6.5	1.8	6.0 (0.65–108.0)	85.2	1.6	20.5 (6.6–44.4)	7.69
Pulling bulband Bulb testing	89	47.8 ± 5.0	1.6	4.4 (0.65–15.0)	81.3	0	15.6 (3.6–34.2)	0
Total	530	45.1 ± 6.6	1.8 ± 1.3	6.0 (0.65^d^-108.0)	86.0	0.9	19.7 (3.6–89.4)	16.7

OEL, Occupational Exposure Limits; BEL, Biological Exposure Limits.

^*a*^Limit of detection for Hg in the air was 1.3μg/m^3^ in this study based on a Chinese standard, i.e., Methods for Determination of Hg and Its Compounds in the Air of Workplace (GBZ/T 160-2004).

^*b*^The OEL for Hg expressed as PC-TWA (permissible concentration-time-weighted average) was 20μg/m^3^ according to the occupational health standard in China, i.e., the Occupational Exposure Limits for Hazardous Agents in the workplace (GBZ 2-2007).

^*c*^The BEL for Hg was 35μg/g·creatinine according to the occupational health standard in China, i.e., the Diagnostic criteria of occupational Hg poisoning (GBZ 89-2007).

^*d*^Values below the detection limit were calculated as 1/2 of the value.

### Risk assessment results

#### Identification of HRs

According to the GHS classification method, the THI for Hg exposure was 75 which was classified as extreme hazard (HR = 1). This classification was consistent with that using both the EPA and Singaporean methods. Further, the HR from the COSHH method was 0.8 which was close to the results from the other three methods.

#### Determination of ERs

Based on the results of Hg concentrations in the air, the exposure was graded at the third level using the AIHA method and the ER was 0.6 (SD 0.00) for each of the three Hg-exposure locations. The same ER value was graded using the Singaporean method for the injection and venting locations.

#### Comparisons of RRs, ERs, and HRs levels using different methods

Based on the Hg concentrations in the workplace air and in urine samples from workers, five assessment methods (except for EPA and Romanian methods) were used to evaluate the exposure and health hazards. For data processing, the RR was applied for comparison of results from the different methods. As shown in Table 3, the highest HR was identified by both the EPA and Singaporean methods but the lowest by both the Australian and Romanian methods. As for ERs, the highest value was assessed by the Australian method while it was at medium value by the Singaporean method and low by the remaining methods. All results showed significant differences (*P* < 0.05) among the methods.

**Table 3:** Comparisons in the risk, exposure and hazard ratio levels with different methods.

**Work types**	**EPA**		**Singaporean**			**COSHH**			**ICMM**			**Australian**			**Romanian**	
	**HR**	**RR**	**HR**	**ER**	**RR**	**HR**	**ER**	**RR**	**HR**	**ER**	**RR**	**HR**	**ER**	**RR**	**HR**	**RR**
Amalgam injection	1.0	1.00 ± 0.00	1.0	0.60 ± 0.00	0.80 ± 0.00	0.8	0.25 ± 0.00	0.60 ± 0.00	0.5	0.33 ± 0.00	0.40 ± 0.00	0.33	1.00 ± 0.00	0.30 ± 0.00	0.29	0.30 ± 0.00
Venting		1.00 ± 0.00		0.60 ± 0.00	0.75 ± 0.09		0.25 ± 0.00	0.60 ± 0.00		0.33 ± 0.00	0.40 ± 0.00		1.00 ± 0.00	0.30 ± 0.00		0.30 ± 0.00
Pulling bulband, bulb testing		0.93 ± 0.16		0.54 ± 0.18	0.74 ± 0.18		0.25 ± 0.00	0.60 ± 0.00		0.36 ± 0.10	0.38 ± 0.11		1.00 ± 0.00	0.30 ± 0.08		0.26 ± 0.08
Total		0.97 ± 0.10^bcdef^		0.58 ± 0.11*	0.76 ± 0.12^acdef^		0.25 ± 0.00	0.60 ± 0.00^abcdf^		0.34 ± 0.06	0.39 ± 0.06^abdce^		1.00 ± 0.00	0.30 ± 0.05^abef^		0.29 ± 0.05^abef^

ER, exposure ratio; HR, hazard ratio; RR, risk ratio.

^*a*^P <0.05 as compared with EPA method.

^*b*^P <0.05 as compared with Singaporean method.

^*c*^P <0.05 as compared with Australian method.

^*d*^P <0.05 as compared with Romanian method.

^*e*^P <0.05 as compared with COSHH Essentials method.

^*f*^P <0.05 as compared with ICMM method.

*P <0.05, as compared with the other methods.

According to the AIHA classification method, the ER of Hg was 0.60.

According to the GHS classification method, the HR of Hg extreme hazard was 1.00.

The results of RR and population distribution were listed in Table 3. The RR was at the highest level using the EPA method (non-carcinogenic effect) with a mean of 0.97 (SD 0.10) while it was close to 0.76 (SD 0.12) using the Singaporean method (*P* < 0.05). The levels were at 0.6 (SD 0.12) and 0.39 (SD 0.06) using the COSHH Essentials and the ICMM methods, respectively. However, the levels were at 0.30 (SD 0.05) and 0.29 (SD 0.05), using the Australian and Romanian methods, respectively, and these levels were significantly different from the other four methods (*P* < 0.05).

In terms of the three different Hg-exposure work positions, the amalgam-injection position was assessed at the highest risk level using the Singaporean method. The bulb pulling/testing was assessed at the lowest risk levels using the EPA, Singaporean, ICMM, and Romanian methods. Furthermore, the risk levels at the injection and venting positions were higher but similar to each other using the EPA, ICMM and Romanian methods. The levels for the three work positions were the same using both the COSHH and Australian methods. There was no significant difference among the three positions (*P* > 0.05).

### Correlation index and levels

**Figure 2:**
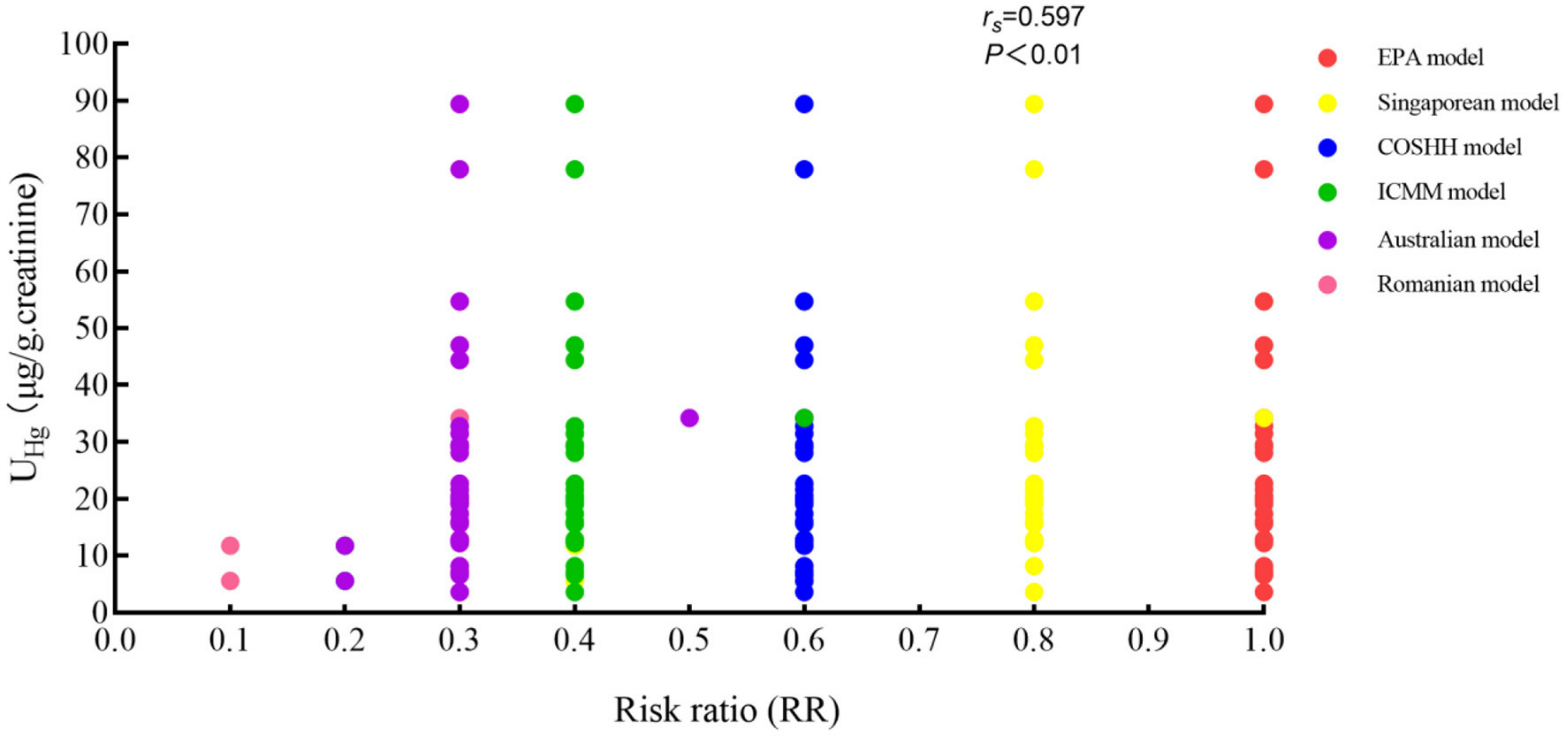
Correlation indices between U_Hg_ and RRs with different OHRA methods.

**Figure 3:**
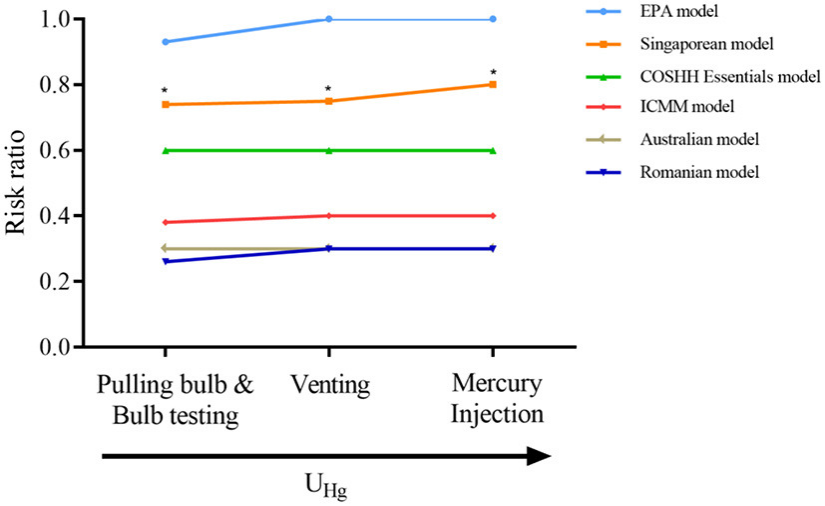
Correlations between U_Hg_ and RRs with different OHRA methods in different work locations. * means *P* < 0.05.

Urinary Hg concentrations were calculated and analyzed using the six evaluation methods. The correlation results are shown in Table 4 and Figure 2. The correlation coefficient was 0.597 using the Singaporean method. The correlation coefficient between the urinary and the aerial concentrations was highly significant at 0.589 (*P* < 0.01). The correlation coefficient among the Romanian, ICMM, Australian methods was 0.830 and that for the EPA and Singaporean methods was 0.619. Both results were statistically significant (*P* < 0.01). In addition, the correlation coefficient between the Hg concentrations and the Singaporean method was 0.722 (*P* < 0.01). With increased RRs values in different work positions, the urinary Hg values showed an upward trend, and the RR value evaluated by the Singaporean method was 0.76 (*P* < 0.01), as shown in Figure 3.

**Table 4:** Correlation analyses between risk ratios and Hg concentrations in air and urine samples.

**OHRA methods**	**EPA**	**Singaporean**	**ICMM**	**Australian**	**Romanian**	**U_Hg_**	**C_Hg_**
EPA	1	–	–	–	–	–	–
Singaporean	0.62**	1	–	–	–	–	–
ICMM	0.83	0.74	1	–	–	–	–
Australian	0.83	0.74	1.00**	1	–	–	–
Romanian	1.00	0.61	0.83**	0.83**	1	–	–
U_Hg_	0.34	0.59**	0.38	0.38	0.34	1	–
C_Hg_	0.44	0.72**	0.53	0.53	0.44	0.58**	1

C_*Hg*_, Hg concentrations in air; U_*Hg*_, Hg concentrations in urine.

**Means P <0.01.

## Discussion

Mercury can be readily detected in human urine after its exposure, therefore urinary Hg has been used as an excellent exposure biomarker for workers (23, 24). Although excessive exposures have been reported, these exposure levels have not been used for assessment of adverse health effects among workers. In one report, 60 % of FLs manufacturing places failed to follow the environmental standards and this failure showed up in 84.6 % of urine samples from workers with excessive Hg-exposure (25). In another report, a regression equation which addressed urine and air Hg concentrations was consistent with our results (26).

Most studies on hazards among FL-exposed workers have been based on environmental sampling of Hg, without biological investigations (7, 12, 16, 27, 28). Since these studies did not connect external and internal doses from the perspective of mathematical statistics, results from these studies might not be highly reliable. As showed in Table 2, the environmental Hg concentrations from 128 sampled sites were all within the OEL, except 1.6% from the venting stations. However, as high as 66.7 % of the urine samples from amalgam-injection workers were above the biological Hg limits. The data indicate that urine-Hg determinations were more useful in detecting excessive exposure and might be more valuable for estimating adverse health effects. Therefore, even based on such information, the workplaces should be modified to bring the BEL for Hg under the occupational health standard of 35 μg/g creatinine level.

It is straight-forward to manage health risks by using risk assessment method when there are no abnormal biological indicators (urinary Hg, et al.). However, practical guidelines or rigorous standards for occupational health risk assessments has not been established yet. Thus, it is prudent to use different risk assessment methods to compare the practices and results. The approach was used for our investigation and our experience also generated recommendations on risk assessment methods for China.

From our study, Table 3 shows that the RRs of Hg exposure based on the EPA, COSHH and Singaporean methods were consistent with results from current risk classifications. Accordingly, the risk levels from higher risk (0.60) to the highest risk (0.97) were indicated by biological indicators. In addition, these three methods used quantitative, semi-quantitative, and qualitative methods, respectively. The RRs of Hg exposure based on the Romanian, Australian and ICMM methods showed the risk levels from low risk (0.29) to below medium risk (0.39) that workers were less likely to have abnormalities in their biological indicators. Table 3 also shows that the RRs from the EPA method were significantly greater than the RRs from the other methods. This indicates that the use of different methods yielded diverse risk assessment results. However, the relatively small RRs based on the Australian, ICMM, and Romanian methods might underestimate risk levels. Therefore, industries should use appropriate methods for more accurate determination of occupational risk than the others, i.e., the Romanian, Australian and ICMM methods.

Table 3 shows that the highest HRs of Hg exposure using both the EPA (1.0) and Singaporean (1.0) methods were the same as the results from the GHS method. This confirms that mercury was extremely dangerous to humans. Furthermore, HRs using the COSHH (0.8) method indicated that mercury was highly harmful to humans while the ICMM (0.5) method indicated moderately toxic. HRs using both the Australian (0.33) and Romanian (0.29) methods showed mercury was less harmful than the other results, and these two methods might underestimate harms to humans.

Unlike other methods, ERs from the Australian (1.00) method were mainly related to the exposure times, therefore this method would indicate that workers were more exposed to Hg. On the other hand, the assessments using the ICMM (0.34) method were greatly affected by subjective factors: as the environmental Hg concentrations were close to the half of the OEL, the method indicated that workers were less likely to be exposed to mercury. The same observations happened to the use of the COSHH (0.25) method. On the other hand, the assessed exposure levels using the Singaporean (0.58) method were consistent with the intrinsic toxicity of Hg and with the concentrations in the air, which were validated using the AIHA method. Consequently, the exposure-risk assessment was accurately determined.

Table 4 shows the correlation analyses using the RRs to test agreements among the different methods. The analyses indicate that the EPA method was only correlated with the Singaporean method but not with the others, and the correlation coefficient reached 0.619 (*P* < 0.05). The results of these two methods had good correlation and might be combined to assess health risks. The correlation coefficient between the ICMM and the Australian methods was 1.000 (*P* < 0.05), while between the ICMM and the Romanian methods was 0.830 (*P* < 0.05), indicating that the results of the three assessments were highly correlated, but all of them might underestimate occupational health risks in the industry. Table 4 also shows that Hg in the urine not only correlated with the Singaporean method (0.597, *P* < 0.05), but also with Hg in the air (0.589, *P* < 0.05), which was also correlated with the Singaporean method (0.722, *P* < 0.05). These results suggest that the Singaporean method was useful in indicating occupational health risks instead of the need for biological indicators of harm. Based on consistency between the assessed risk and the urinary levels of Hg, our investigation indicates that the Singaporean method was better than the others for identifying exposure-related health risk for the FL workers.

The OHRA methods exhibited a diverse combination of different evaluation indicators in high and low ranking, suggesting that several factors should be considered when multiple evaluation methods were used to perform OHRA. Both the COSHH and Singaporean methods were regarded as more practical ones since they provided detailed control strategies to reduce potential occupational health risks. Meanwhile, the qualitative and semi-quantitative methods were easy to manage in terms of operability. Therefore, the Singaporean, COSHH, and EPA methods received higher total scores than the others after taking all the evaluation indicators into consideration. This suggests that the latter methods were more appropriate for OHRA applications because of their advantages, especially in reliability.

From the six occupational health risk assessment methods, our study discovered that the EPA, COSHH and Singaporean methods were able to identify the high-risk positions according to urinary rather than environmental Hg concentrations. Therefore, our investigation provided strong data in support of our recommendations. Thus, risk assessments using different risk methods would produce highly different results which were consistent with the pros and cons for each method.

Although our study was conducted according to our design, it has three main limitations. First, the applicability of some assessment methods was based on the Chinese occupational standards. Differences of multiple occupational standards from different countries could lead to differences in evaluation of health risks. Second, the lack of evidence for organ damage from excessive exposure to mercury would reduce the confidence on assessed health risks. Lastly, the applicability of Singapore model for low-dose Hg exposures and quantitative variables from such exposures remains to be validated. In further studies, detailed understanding of the types of occupational hazards, technological processes and job characteristics, precisions in grouping of Hg-exposed workers, are needed for more accurate evaluations.

## Data availability statement

The raw data supporting the conclusions of this article will be made available by the authors, without undue reservation.

## Ethics statement

The studies involving human participants were reviewed and approved for research from Hangzhou Hospital for Prevention and Treatment of Occupational Disease (IRB: 2020-001). The patients/participants provided their written informed consent to participate in this study.

## Author contributions

X-yR collected and analyzed the data and wrote the manuscript. T-sW, LZ, and J-mY contributed to the study design and manuscript preparation. Y-pS and HF contributed to data collection and field investigation. S-wT analyzed and processed laboratory samples. M-bZ conceived the ideas and supervised the study. All authors contributed to the article and approved the submitted version.

## Conflict of interest

The authors declare that the research was conducted in the absence of any commercial or financial relationships that could be construed as a potential conflict of interest.

## Publisher's note

All claims expressed in this article are solely those of the authors and do not necessarily represent those of their affiliated organizations, or those of the publisher, the editors and the reviewers. Any product that may be evaluated in this article, or claim that may be made by its manufacturer, is not guaranteed or endorsed by the publisher.

## Abbreviations

OHRA: occupational health risk assessment
EPA: Environmental Protection Agency
ICMM: International Council on Mining and Metals
COSHH: The Control of Substances Hazardous to Health
RR: risk ratio
BEL: biological exposure limit
FL: fluorescent lamp
ACGIH: American Conference of Governmental Industrial Hygienists
OEL: Occupational exposure limit
PPE: personal protective equipment
PC-TWA: permissible concentration-time weighted average
U-Hg: urinary Hg
HR: hazard ratio
ER: exposure ratio
IARC: International Agency for Research on Cancer
THI: Toxicant Hazardous Index
GHS: Globally Harmonized System of Classification and Labeling of Chemicals
LC_50_: median lethal concentration
LD_50_: median lethal does
HSDB: US Hazardous Substances Database
RTECS: Registry of Toxic Effects of Chemical Substances
AIHA: American Industrial Hygiene Association.
